# Consumer‐grade biosensor validation for examining stress in healthcare professionals

**DOI:** 10.14814/phy2.14454

**Published:** 2020-06-02

**Authors:** Luke Hopkins, Benjamin Stacey, David B. T. Robinson, Osian P. James, Christopher Brown, Richard J. Egan, Wyn G. Lewis, Damian M. Bailey

**Affiliations:** ^1^ Health Education and Improvement Wales’ School of Surgery Nantgarw UK; ^2^ Department of Surgery Morriston Hospital Swansea UK; ^3^ Neurovascular Research Laboratory Faculty of Life Sciences and Education University of South Wales Pontypridd UK

**Keywords:** biosensor, exercise, physiological, stress, validation

## Abstract

**Introduction:**

A high prevalence of stress and burnout has been reported among healthcare professionals; however, the current tools utilized to quantify such metrics are not in keeping with doctors’ busy lifestyles, and moreover do not comply with infection prevention policies. Given that increased stress can subsequently impact both the healthcare profession and the patient in care, this study aimed to assess the validity of a wearable biosensor to monitor and manage stress experienced by healthcare professionals.

**Methods:**

In all, 12 healthy, male volunteers completed an incremental exercise protocol to volitional exhaustion, which aimed to induce physiological stress in a graded manner. A wearable consumer‐grade biosensor (Vital Scout, VivaLNK, Inc.) was used to measure stress, energy expenditure, respiration rate, and activity throughout the exercise protocol. These variables were validated against online breath‐by‐breath analysis (MedGraphics Ultima Series).

**Results:**

When compared against online “gold standard” measurements, the Vital Scout biosensor demonstrated a high level of accuracy to measure energy expenditure (*r* = .776, *p* < .001) and respiration rate (*r* = .744, *p* < .001). The $\dot{V}O_{2}$ increase observed during the incremental exercise test was associated with the Vital Scout biosensor's measurement of activity (*r* = .777, *p* < .001). In contrast, there was a poor relationship between the changes in $\dot{V}O_{2}$ and the Vital Scout biosensor's ability to detect stress (*r* = −.195, *p* = .013).

**Conclusion:**

The Vital Scout biosensor provided an accurate assessment of energy expenditure and respiration when compared to the “gold standard” assessment of these parameters. Biosensors have the potential to measure stress and deserve further research in the peri‐hospital environment.

## Graphical abstract

Increased stress can impact both the healthcare profession and the patient in care, this study aimed to assess the validity of a wearable biosensor to monitor and manage stress experienced by healthcare professionals. A wearable consumer‐grade biosensor was used to measure stress, energy expenditure, respiration rate and activity throughout the exercise protocol. These variables were validated against online breath‐by‐breath analysis. When compared against online ‘gold standard’ measurements, the biosensor demonstrated a high level of accuracy to measure energy expenditure (*r* = .776, *p* < .001) and respiration rate (*r* = .744, *p* < .001).

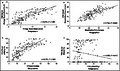

## 1 INTRODUCTION

Evolution has favored compensatory mechanisms to allow humans to facilitate short‐term stressors and while in small, periodical doses, this rarely has implications for health. However, excessive exposure to stress has the potential to manifest into anxiety, depression, substance abuse, and often suicide. Undeniably, training for and practicing surgery places a significant amount of physiological and psychological stress on healthcare professionals (Robinson et al., 2019). While this is clearly detrimental to the quality of life of a surgeon, an increase in peri‐operative stress also has the potential to adversely affect both technical and non‐technical performance (Grantcharov, Boillat, Elkabany, Wac, & Rivas, 2019), exposing patients to greater risk of malpractice. Given that younger surgeons are more susceptible to stress and that “burnout” has been strongly associated with the desire to pursue early retirement (Campbell, Sonnad, Eckhauser, Campbell, & Greenfield, 2001), this is likely an issue that will continue to persist in the years to come.

Stress can be measured using a variety of assessment tools including psychological surveys, biological markers (cortisol) and more recently, the assessment of physiological parameters including heart rate variability (Arora et al., 2010). While utilizing some of these tools to assess stress among surgeons would be very impractical, the development of wearable biosensors would permit continual assessment of heart rate variability, an in‐direct measurement of stress, during both live clinical procedures and out‐of‐hours monitoring, while recuperating. However, it is crucial that the parameters measured by the wearable biosensor are evaluated for accuracy first. Therefore, to assess the efficacy of the wearable biosensor, the aim of the present study was to first, compare the primary measurements of respiration rate and energy expenditure against data obtained from online breath‐by‐breath analysis and second, evaluate its performance during an incremental exercise test to determine whether physical activity‐induced changes influence the stress measurement.

## 2 METHODS

### 2.1 Ethical approval

Ethical approval was provided by the University of South Wales Research Ethics Committee (201712BS01) as part of a larger investigation. All procedures were carried out in accordance with the Declaration of Helsinki of the World Medical Association.

### 2.2 Participants

In all, 12 healthy, male volunteers were recruited into the study. They were 29 ± 4 years old, with a stature of 1.76 ± 0.05 m and body mass of 82 ± 14 kg. None of the participants were taking prescribed medications from their medical practitioner, in particular drugs with a chronotropic action on the cardiovascular system.

### 2.3 Incremental exercise test

An incremental exercise test to volitional exhaustion was conducted using an electromagnetically braked cycle ergometer (Lode) to induce physiological stress in a graded manner. The initial workload was set at 70 Watts (W) and was increased by 30 W/minute until volitional exhaustion. Expired gas fractions, minute ventilation respiration rate, and oxygen consumption ( $\dot{V}O_{2}$) were measured via breath‐by‐breath online gas analysis (MedGraphics Ultima Series) and used to calculate indirect calorific energy expenditure.

### 2.4 Vital Scout biosensor

The VitalScout wearable biosensor (VivaLNK, Inc.) was used according to the manufacturer's instructions with data collected using the VitalScout app (v2.0.6) connected to a smartphone via Bluetooth^®^. The wearable patch was positioned on the chest in line with the heart. The biosensor contains a two‐lead ECG and accelerometer, which allowed for the measurement of heart rate, respiratory rate, calorific energy expenditure, activity, and stress. Heart rate variability was used to produce stress indices analogous to sympathetic activation.

### 2.5 Statistical analysis

Data were analyzed using the Statistical Package for Social Sciences (IBM SPSS Statistics Version 25.0). Shapiro–Wilk *W* tests confirmed that all datasets were normally distributed. Correlation between variables were assessed using the Pearson correlation coefficient. Significance for all two‐tailed tests was established at *p *< .05.

## 3 RESULTS

**FIGURE 1:**
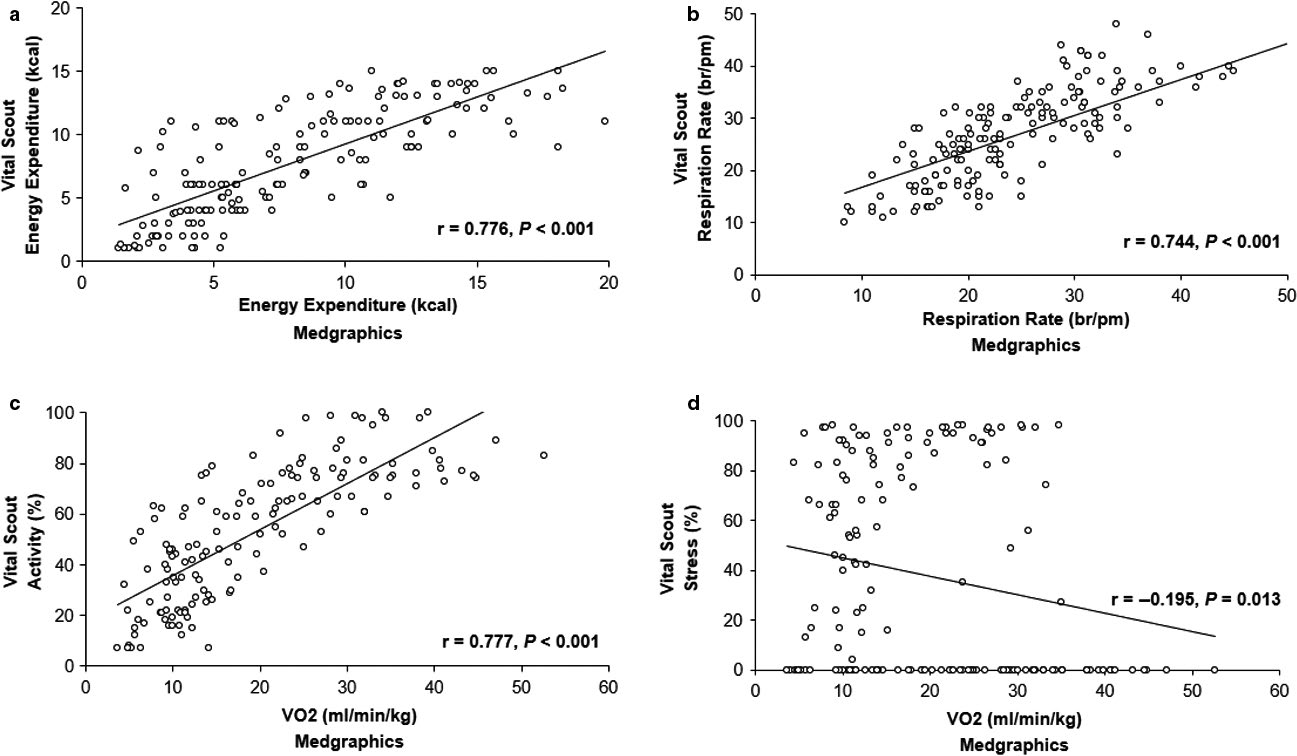
A comparison of physiological variables obtained from the Vital Scout and Medgraphics during an incremental exercise test. (a) energy expenditure; (b) respiration rate; (c) activity levels obtained from the Vital Scout versus $\dot{V}O_{2}$ from the Medgraphics; and (d) stress levels obtained from the Vital Scout versus $\dot{V}O_{2}$ from the Medgraphics

When compared against “gold standard” online measurements, the Vital Scout biosensor demonstrated a high level of accuracy to measure energy expenditure (*r* = .776, *p* < .001) and respiration rate (*r* = .744, *p* < .001) (Figure 1). Additionally, the increase in $\dot{V}O_{2}$ during the incremental exercise test, as measured with the Medgraphics, correlated strongly with the Vital Scout biosensor's measurement of activity (*r* = .777, *p* < .001) (Figure 1). Lastly, there was a poor relationship between the changes in $\dot{V}O_{2}$ and the Vital Scout biosensor's ability to detect stress (*r* = .195, *p* = .013) (Figure 1), with a large number of data points (75 out of 161) registering stress as zero during the incremental exercise test.

## 4 DISCUSSION

The wearable consumer‐grade biosensor provided an accurate and valid assessment of the physiological changes seen during an incremental exercise challenge. The poor correlation seen between $\dot{V}O_{2}$ and the stress index recorded by the biosensor suggests that the biosensor can accurately determine when increased autonomic activity is due to exercise, as opposed to psychological stress.

Measuring stress in healthcare professionals in a hospital environment is challenging. Completion of psychological surveys during or following stressful events is time‐consuming and may interfere with patient care (Campbell et al., 2001). Additionally, the use of biochemical assays is extremely costly and would require procurement and storage of human tissue. The use of HRV in the occupational setting has partly been limited by the costs of equipment such as Holter monitors or the significant limitations imposed by infection control and theatre sterility regulation, precluding smartwatch‐based measurements.

The biosensor employed in the present study will allow for continuous monitoring throughout all aspects of healthcare professionals’ lives, over a period of 5 days without the need to re‐charge. Weenk et al. (2018) reported that while the operating theatre presented with the most stressful environment for both consultant surgeons and surgeons in training alike, it is clearly apparent that non‐operative technical tasks, including administrative responsibilities, out‐patient clinic consultations, in‐patient ward care, teaching medical students as well as domestic life also contributed to the stress and burnout load. Undoubtedly, the quality of recovery time after work is also crucial, but regrettably receives scant regard.

The long‐term effects of stress on health are thought to be partly due to impaired physical and psychological recovery. Healthcare professionals’ lives are associated with notoriously long‐working hours and despite legally mandated working‐hour restrictions, all grades of surgeons continue to report chronic fatigue and poor sleep quality (Brown, Abdelrahman, Lewis, Pollitt, & Egan, 2019; Coleman et al., 2019). Brown et al. (2019) found that it took five full days for the sleep patterns of surgeons in training to return to normal sleep after night shift work. This has implications for the design of rotas that need to be planned to allow for sufficient recovery prior to clinical duties resuming following night shifts. Among Emergency Physicians and Obstetricians, working night shifts was found to be associated with a decrease in heart rate variability, commensurate with an increase in stress, when compared with working day time on‐call shifts (Amirian, Toftegård Andersen, Rosenberg, & Gögenur, 2014; Dutheil et al., 2012). Given that there is also evidence of an increased cardiovascular morbidity found in night shift workers (Vetter et al., 2016), continual monitoring of a healthcare professional's stress‐related variables is eminently justified.

## 5 CONCLUSIONS

In conclusion, the VitalScout biosensor provided an accurate assessment of physiological parameters during an incremental exercise challenge designed to simulate incremental physiological stress. Furthermore, the stress algorithm appears to be reliable owing to the poor relationship with increases in physical activity. The VitalScout biosensor provides sufficient accuracy to merit further research within the hospital environment. Owing to the exponential increase in stress and burnout among healthcare professionals and the subsequent implications for patient care, it is essential that occupational stress is measured continuously and accurately, before implementation strategies can be in place to manage and improve the quality of life for healthcare professionals and patients alike.

## CONFLICT OF INTEREST

The authors declare no conflict of interest.

## AUTHOR CONTRIBUTION

LH, BS, RJE, WGL, and DMB contributed to the conception and design of the study. BS, LH, DBTR, OPJ, and CB assisted with the measurements. LH, BS, and DMB analyzed the data. LH, BS, WGL and DMB wrote the first draft of the manuscript. All authors read and approved the final manuscript.
